# Restrictions to HIV-1 replication in resting CD4^+^ T lymphocytes

**DOI:** 10.1186/1742-4690-10-S1-O25

**Published:** 2013-09-19

**Authors:** Oliver Fackler

**Affiliations:** 1 University of Heidelberg, Germany

## 

CD4^+^ T lymphocytes represent the main target cell population of human immunodeficiency virus (HIV). In an activated state, CD4^+^ T cells residing in lymphoid organs are a major reservoir of ongoing HIV-1 replication in infected individuals. In contrast, resting CD4^+^ T cells are highly resistant to productive HIV-1 infection, yet are massively depleted during disease progression and pose a substantial latent reservoir for the virus *in vivo.* Barriers preventing replication HIV-1 in resting CD4^+^ T cells include a rigid layer of cortical actin and, early after HIV-1 entry, a block that limits reverse transcription of incoming viral RNA genomes. Defining the molecular bases of these restrictions has remained one of the central open questions in HIV research. Recent advances unraveled mechanisms by which HIV-1 bypasses the entry block and established the host cell restriction factor SAMHD1 as a central determinant of the cellular restriction to HIV-1 reverse transcription in resting CD4^+^ T cells. Our current molecular and pathophysiological understanding of the multi-faceted interactions of HIV-1 with resting CD4^+^ T lymphocytes will be discussed.

